# Identification of potentially relevant metals for the etiology of autism by using a Bayesian multivariate approach for partially censored values

**DOI:** 10.1038/s41598-023-38780-9

**Published:** 2023-08-03

**Authors:** Bertil Wegmann, Patricia Tatemoto, Stefan Miemczyk, Johnny Ludvigsson, Carlos Guerrero-Bosagna

**Affiliations:** 1 Division of Statistics and Machine Learning, Department of Computer and Information Science, Linköping University, 581 83 Linköping, Sweden; 2 Center for Comparative Studies in Sustainability, Health and Welfare, Department of Veterinary Medicine and Animal Health, School of Veterinary Medicine and Animal Science, FMVZ, University of São Paulo, Pirassununga, São Paulo 13635-900 Brazil; 3 Avian Behavioral Genomics and Physiology Group, Department of Physics, Chemistry and Biology (IFM), Linköping University, 581 83 Linköping, Sweden; 4 Crown Princess Victoria Children’s Hospital and Division of Pediatrics, Department of Biomedical and Clinical Sciences, Linköping University, Linköping, Sweden; 5 Physiology and Environmental Toxicology Program, Department of Organismal Biology, Uppsala University, 75236 Uppsala, Sweden

**Keywords:** Autism spectrum disorders, Diagnostic markers

## Abstract

Heavy metals are known to be able to cross the placental and blood brain barriers to affect critical neurodevelopmental processes in the fetus. We measured metal levels (Al, Cd, Hg, Li, Pb and Zn) in the cord blood of newborns and in the serum of the same children at 5 years of age, and compared between individuals with or without (controls) autism spectrum disorder (ASD) diagnosis. The samples were from a biobank associated with the All Babies in Southeast Sweden (ABIS) registry. We proposed a Bayesian multivariate log-normal model for partially censored values to identify potentially relevant metals for the etiology of ASD. Our results in cord blood suggest prenatal Al levels could be indicative of later ASD incidence, which could also be related to an increased possibility of a high, potentially toxic, exposure to Al and Li during pregnancy. In addition, a larger possibility of a high, potentially beneficial, exposure to Zn could occur during pregnancy in controls. Finally, we found decisive evidence for an average increase of Hg in 5-year-old ASD children compared to only weak evidence for controls. This is concordant with previous research showing an impaired ability for eliminating Hg in the ASD group.

## Introduction

Autism spectrum disorders (ASD) represent a group of disorders that includes five diagnostic subtypes^1^: (i) Autism, (ii) pervasive developmental disorders not otherwise specified (PDD-NOS), (iii) Rett’s disorder, (iv) child disintegrative disorder, and (v) Asperger’s disorder. ASD are usually regarded as neurodevelopmental disorders in which affected children develop impairments in communication and social interaction, together with restricted and repetitive behaviors^1^. ASD are estimated to affect 1 in 100 children worldwide, according to the World Health Organization^2^. Both genetic and environmental factors are shown to contribute to the pathogenesis of ASD^1^. Findings from identical twin and sibling studies indicate a strong genetic component contributing to the prevalence of ASD^3,4^. Since the first diagnosis of ASD, a 5–10 fold increased prevalence of ASD has been reported^5^. This drastic increase can only partially be attributed to improved diagnostics^1^.

In recent years, neurodevelopmental disorders in general, and ASD in particular, have been related to heavy metals exposure^6–8^, particularly during early development^6,9^. In a recent review,^10^ emphasize that although studies on toxic elements have been largely limited by their design, enough evidence exists associating heavy metals (notably mercury and lead) and ASD to justify research on the topic.

Parental exposures occurring before or during pregnancy could influence the capacity of children to detox heavy metals, leading to their accumulation, which could then affect brain development. Heavy metals are known to be able to cross the placental and blood brain barriers and then potentially affect critical neurodevelopmental processes in the fetus^11^. Toxicity of heavy metals can occur via different molecular mechanisms, such as the interaction of metal ions with neurotransmitters, receptors, ion pumps, biological calcium, enzymes as well as functional groups of certain amino acids^12,13^.

In the present paper, we have investigated the presence of heavy metals in the cord blood of newborns and then in the serum of these children at 5 years of age. We compared samples from children with ASD diagnosis to samples from children without ASD diagnosis (controls) to identify whether some of these metals could be of relevance for ASD etiology. The study employed samples from a biobank related to the All Babies in South Sweden (ABIS) registry. Levels of aluminum (Al), cadmium (Cd), mercury (Hg), lithium (Li), lead (Pb) and zinc (Zn) were measured both in the cord blood of newborns and in their serum at 5 years of age. For the identification of the heavy metals that could be relevant for the etiology of autism, we have employed a Bayesian multivariate log-normal model that accounts for censored metal values in each of the experimental groups.

## Material and methods

All methods were performed in accordance with the relevant guidelines and regulations.

### Origin of samples

The samples are part of the biobank associated to the ABIS (All Babies in Southeast Sweden) registry. The samples are stored at the Division of Pediatrics of the Department of Biomedical and Clinical Sciences, Linköping University. The original goal of the ABIS study was to investigate the emergence of Type 1 diabetes and other immune mediated diseases in Swedish children^14^. Informed consent was obtained from the legal guardians of all participating children. In this context cord blood, breastmilk, hair of the mother was collected at birth, while blood, urine, stool, and hair were obtained from children at the ages of 1, 3 and 5 years. The ABIS cohort comprised male and female children born between Oct 1997 and Oct 1999 in the Swedish counties of Östergötland, Öland, Blekinge, and Småland, which are followed prospectively. Using this cohort, we have recently reported associations between blood heavy metals in children and Type 1 diabetes^15^ and autoimmunity^16^.

### Database employed for diagnosis

The categorization of the samples as coming from children with or without ASD diagnosis was performed by different medical doctors across Sweden and incorporated into The Swedish National Patient Register. This population-based register was launched in 1964 and is currently maintained by the Swedish National Board of Health and Welfare (http://www.socialstyrelsen.se/english). Over 99% of all somatic and psychiatric hospital discharges, as well as outpatient visits from both private and public caregivers, are recorded in this register. The recorded items are based on the International Classification of Diseases (ICD) codes, and associate with the personal identity number (a unique 10-digit number) assigned to all Swedish residents (http://www.socialstyrelsen.se/english). Over 99% of all somatic and psychiatric hospital discharges, as well as outpatient visits from both private and public caregivers, are recorded in this register.

### Analysis of metal levels

The samples were analyzed for the concentration of aluminum (Al), cadmium (Cd), mercury (Hg), lithium (Li), lead (Pb) and zinc (Zn). For cord blood, 20 samples were randomly selected and analyzed from ASD diagnosed children and 40 from the control group. For the serum from 5-year-old children, 11 samples were analyzed from ASD diagnosed children and 24 from the control group. The analysis of metal levels was performed by ALS Scandinavia AB (Luleå, Sweden) using the ‘ultrasensitive inductively coupled plasma sector field mass spectrometry method’ (ICP-SFMS)^17^ after acid digestion with HNO3, according to the standards ISO 17294-1^18^ and ISO 17294-2^19^, and the EPA Method 200.8^20^. Most metal levels were lower than the detection level of each metal, which refers to censored metal levels from above, and a few metal values were not recorded (missing data). In the next section, we incorporate these features of our data into our Bayesian modeling of metal values.

### Ethical approval

The study was approved by the Research Ethical committee, Linköping university (Dnr: M138-09 and Dnr: 2016/515-31).

## Bayesian modeling of metal values

The Bayesian approach formulates a prior distribution for all model parameters, and then updates this prior distribution with observed data through the likelihood function to a posterior distribution. The goal of a Bayesian analysis is to make inference on the posterior distribution of all model parameters.

### Likelihood function

Let $x=\left( x_{1},x_{2},\ldots ,x_{J}\right)$ be the vector of values for the *J* different metals of any individual in one of the groups. Assume for each of the groups that

1 $$\begin{aligned} \ln x\sim N\left( \mu ,\Sigma \right) , \end{aligned}$$

where $\mu$ is the mean vector and $\Sigma$ is the covariance matrix for that group. This implies that *x* follows a multivariate log-normal distribution with mean for each metal *j* given by

$$\begin{aligned} \mu _{xj}=\exp \left[ \mu _{j}+\frac{1}{2}\Sigma _{jj}\right] \end{aligned}$$

and covariance matrix for elements *j* and *k* as

$$\begin{aligned} \Sigma _{xjk}=\exp \left[ \mu _{j}+\mu _{k}+\frac{1}{2}\left( \Sigma _{jj}+\Sigma _{kk}\right) \right] \left( \exp \left[ \Sigma _{jk}\right] -1\right) \end{aligned}$$

for any types of metals *j* and *k*. Let $y=\ln x$ and partition *y* as $y=\left( y_{1},y_{2}\right) ,$ where $y_{1}$ is the vector of observed values and $y_{2}$ is the vector of censored values for any individual *i*. Similarly, partition the mean vector $\mu =\left( \mu _{1},\mu _{2}\right)$ and covariance matrix

$$\begin{aligned} \Sigma =\left( \begin{array}{cc} \Sigma _{11} &{} \Sigma _{12}\\ \Sigma _{21} &{} \Sigma _{22} \end{array}\right) , \end{aligned}$$

where $\Sigma _{12}=\Sigma _{21}$ by symmetry. Then, it follows that the marginal distribution of $y_{1}$ is given by

$$\begin{aligned} y_{1}\sim N\left( \mu _{1},\Sigma _{11}\right) , \end{aligned}$$

and the conditional distribution of $y_{2}|y_{1}$ becomes^21^

$$\begin{aligned} y_{2}|y_{1}\sim N\left( \mu _{2}+\Sigma _{21}\Sigma _{11}^{-1}\left( y_{i1}-\mu _{1}\right) ,\Sigma _{22}-\Sigma _{21}\Sigma _{11}^{-1}\Sigma _{12}\right) . \end{aligned}$$

Let $f_{y_{1}}\left( y_{1}\right)$ be the probability density function of $y_{1}$and let $F_{y_{2}|y_{1}}\left( c_{2}\right)$ denote the cumulative distribution function of $y_{2}|y_{1}$ evaluated at $c_{2}$. The likelihood function ${\mathscr {L}}_{i}$ for any individual *i* becomes ${\mathscr {L}}\left( \mu ,\Sigma |y_{i1},y_{i2}\right) =f_{y_{1}}\left( y_{i1}|\mu _{i},\Sigma _{i}\right) F_{y_{2}|y_{1}}\left( c_{i2}|\mu _{i},\Sigma _{i}\right) ,$ where the elements of $y_{2}$ are censored at $c_{i2}$ from above. Then, the likelihood function for *n* individuals in a group becomes

2 $$\begin{aligned} {\mathscr {L}}\left( \mu ,\Sigma |y_{1},y_{2}\right) =\prod _{i=1}^{n}f_{y_{1}}\left( y_{i1}|\mu _{i},\Sigma _{i}\right) F_{y_{2}|y_{1}}\left( c_{i2}|\mu _{i},\Sigma _{i}\right) . \end{aligned}$$

### Bayesian inference

The posterior distribution of $\left( \mu ,\Sigma \right)$ is given through Bayes’ theorem by

$$\begin{aligned} p\left( \mu ,\Sigma |y_{1},y_{2}\right) \propto {\mathscr {L}}\left( \mu ,\Sigma |y_{1},y_{2}\right) p\left( \mu ,\Sigma \right) , \end{aligned}$$

where ${\mathscr {L}}\left( \mu ,\Sigma |y_{1},y_{2}\right)$ is the likelihood function in Eq. (2) above and $p\left( \mu ,\Sigma \right)$ is the prior distribution of $\left( \mu ,\Sigma \right)$. Following^22^, we estimate the posterior distribution of $\left( \mu ,\Sigma \right)$ using the default Markov chain Monte Carlo (MCMC) algorithm for $K=1$ mixture component in the R package mixAK. This R package allows for censored values $y_{2}$, which is an inherent feature of our data (see Section *Analysis of metal levels*), where these values are lower than the detection level of the measuring device. We also use the feature of interval-censored values in mixAK for the non-recorded values (missing data) specified on reasonable intervals.

As a standard of comparison, we use the weakly informative prior distributions in mixAK. Weakly informative priors are often considered in Bayesian inference for two reasons: to obtain stable estimation of the posterior and, at the same time, carry very little prior information in such a way that these priors are essentially non-informative prior distributions. Convergence of the MCMC algorithm in mixAK is monitored using the convergency measures ${\hat{R}}$ and ${\hat{n}}_{eff}$ in^23^. The convergency measure ${\hat{R}}$ is approximately equal to the square root of the variance of the mixture of all the chains divided by the average within-chain variance and ${\hat{n}}_{eff}$ is the number of efficient draws as an estimated number of independent posterior draws. We estimated all models such that ${\hat{R}}<1.1$ and ${\hat{n}}_{eff}>100$. This suggested good convergence to the posterior with well-mixed chains.

### Classification

We obtain a posterior distribution for the probability that each observation $y_{i}$ for individual *i* belongs to one of the two groups using a Leave-One-Out Cross-Validation (LOOCV) procedure. Given each observation $y_{i}$, we estimate the model for each group using all other observations $y_{-i}$ than $y_{i}$ as training data. The probability of being in the autism group (here class $C=c_{1}$) instead of the control group (here class $C=c_{2}$) for an individual *i* can be written by reverting Bayes Theorem as (similarly to naive Bayes classification, see e.g.^24^)

$$\begin{aligned}{} & {} p\left( C_{i}=c_{1}|y_{i},y_{-i}\right) =\frac{p\left( y_{i}|y_{-i},C_{i}=c_{1}\right) p\left( C_{i}=c_{1}|y_{-i}\right) }{p\left( y_{i}|y_{-i}\right) }\\{} & {} =\frac{p\left( C_{i}=c_{1}\right) \int p\left( y_{i}|\mu _{c_{1}},\Sigma _{c_{1}}\right) p\left( \mu _{c_{1}},\Sigma _{c_{1}}|y_{-i}\right) d\mu _{c_{1}}d\Sigma _{c_{1}}}{\sum _{j=1}^{2}p\left( C_{i}=c_{j}\right) \int p\left( y_{i}|\mu _{c_{j}},\Sigma _{c_{j}}\right) p\left( \mu _{c_{j}},\Sigma _{c_{j}}|y_{-i}\right) d\mu _{c_{j}}d\Sigma _{c_{j}}}. \end{aligned}$$

We assume equiprobable classes apriori such that $p\left( C_{i}=c_{1}|y_{-i}\right) =p\left( C_{i}=c_{2}|y_{-i}\right) =\frac{1}{2}$ for any observation *i*, which simplifies the probability expression to

$$\begin{aligned} p\left( C_{i}=c_{1}|y_{i},y_{-i}\right) =\frac{\int p\left( y_{i}|\mu _{c_{1}},\Sigma _{c_{1}}\right) p\left( \mu _{c_{1}},\Sigma _{c_{1}}|y_{-i}\right) d\mu _{c_{1}}d\Sigma _{c_{1}}}{\sum _{j=1}^{2}\int p\left( y_{i}|\mu _{c_{j}},\Sigma _{c_{j}}\right) p\left( \mu _{c_{j}},\Sigma _{c_{j}}|y_{-i}\right) d\mu _{c_{j}}d\Sigma _{c_{j}}}. \end{aligned}$$

In addition, the odds for an individual *i* being in the control group also simplifies to

$$\begin{aligned} \frac{p\left( C_{i}=c_{2}|y_{i},y_{-i}\right) }{p\left( C_{i}=c_{1}|y_{i},y_{-i}\right) }=\frac{p\left( y_{i}|y_{-i},C_{i}=c_{2}\right) }{p\left( y_{i}|y_{-i},C_{i}=c_{1}\right) }. \end{aligned}$$

## Results

The results of the model parameters $\mu$ and $\Sigma$ in Eq. (1) are shown in the first subsection for the data on metal values in the cord blood. Differences in $\mu$ and $\Sigma$ between the ASD and control groups for 5-year-olds are shown in the second subsection. Group comparisons between metal values in the cord blood and in 5-year-olds are shown in the third subsection. Finally, classification results are presented in the fourth subsection. Throughout this section, let $\mu _{A}=E_{A}\left[ x\right] ,\mu _{C}=E_{C}\left[ x\right]$ be the expected values of *x* and $\sigma _{A}=\sqrt{Var_{A}\left[ x\right] },\sigma _{C}=\sqrt{Var_{C}\left[ x\right] }$ be the standard deviations of *x* for the ASD and control groups, respectively.

### Metal values in the cord blood

**Figure 1:**
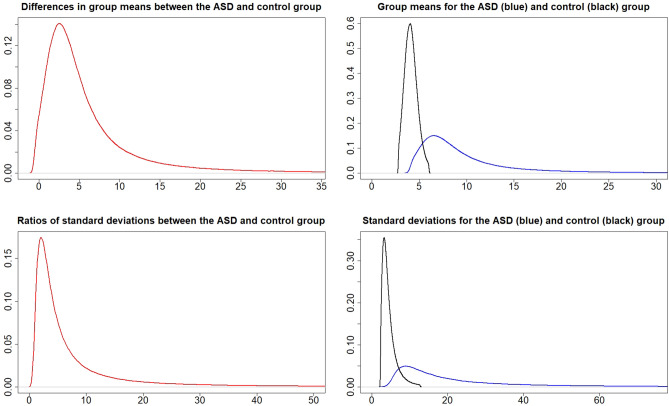
Posterior distributions of $\mu _{A}-\mu _{C}$ (top left), $\mu _{A}$ and $\mu _{C}$ (top right), $\frac{\sigma _{A}}{\sigma _{C}}$ (bottom left), and $\sigma _{A}$ and $\sigma _{C}$ (bottom right) for metal Al.

**Figure 2:**
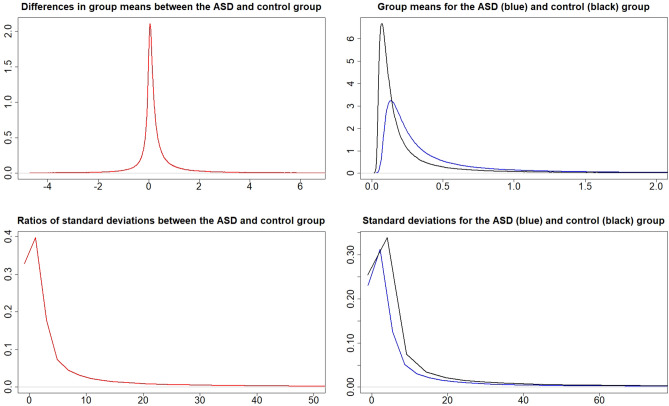
Posterior distributions of $\mu _{A}-\mu _{C}$ (top left), $\mu _{A}$ and $\mu _{C}$ (top right), $\frac{\sigma _{A}}{\sigma _{C}}$ (bottom left), and $\sigma _{A}$ and $\sigma _{C}$ (bottom right) for metal Cd.

**Figure 3:**
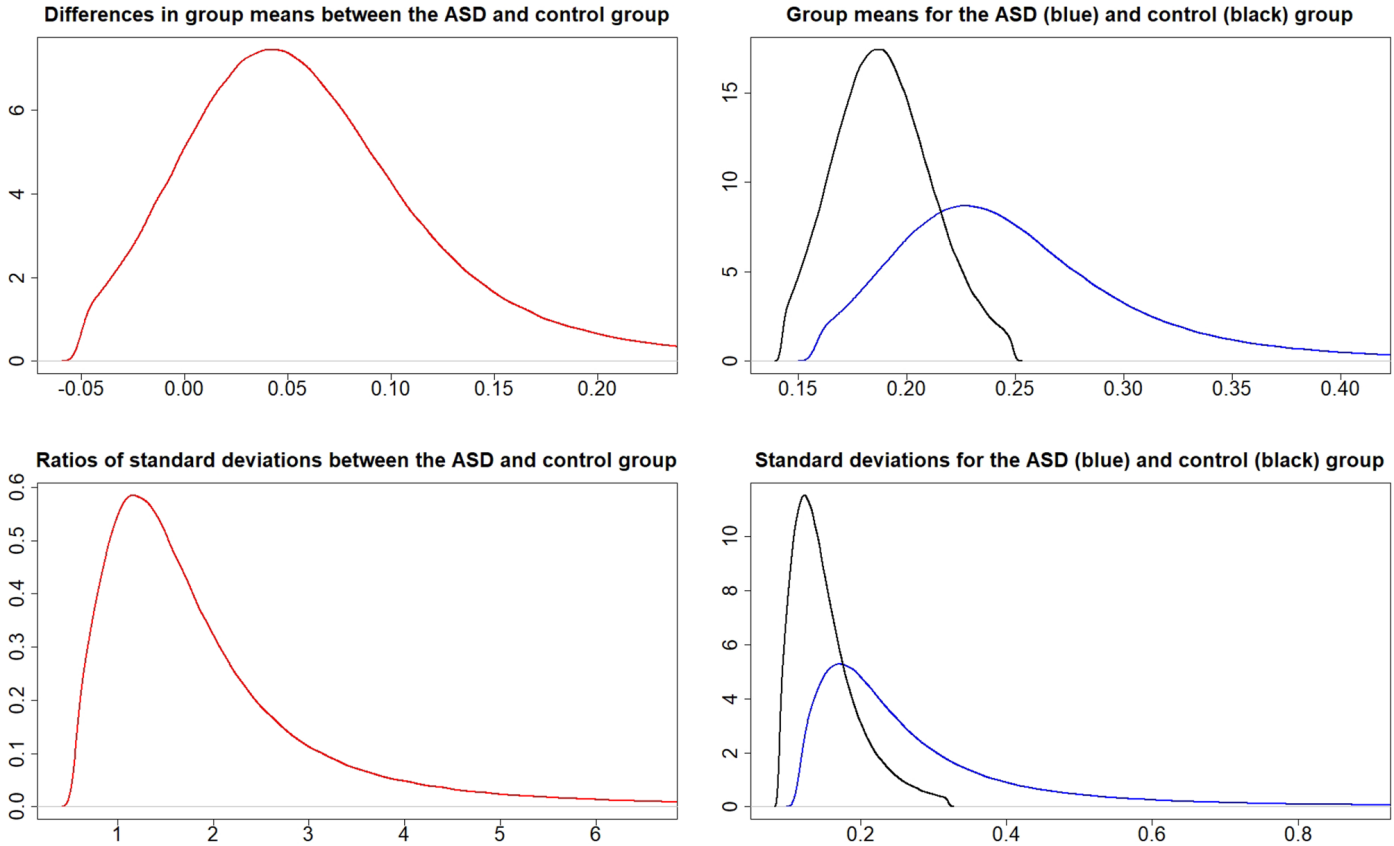
Posterior distributions of $\mu _{A}-\mu _{C}$ (top left), $\mu _{A}$ and $\mu _{C}$ (top right), $\frac{\sigma _{A}}{\sigma _{C}}$ (bottom left), and $\sigma _{A}$ and $\sigma _{C}$ (bottom right) for metal Hg.

**Figure 4:**
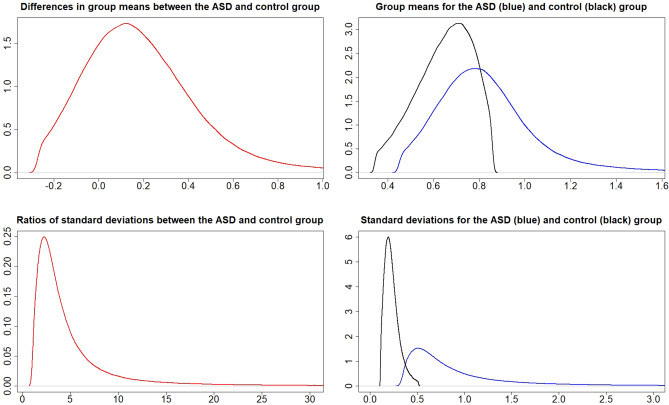
Posterior distributions of $\mu _{A}-\mu _{C}$ (top left), $\mu _{A}$ and $\mu _{C}$ (top right), $\frac{\sigma _{A}}{\sigma _{C}}$ (bottom left), and $\sigma _{A}$ and $\sigma _{C}$ (bottom right) for metal Li.

**Figure 5:**
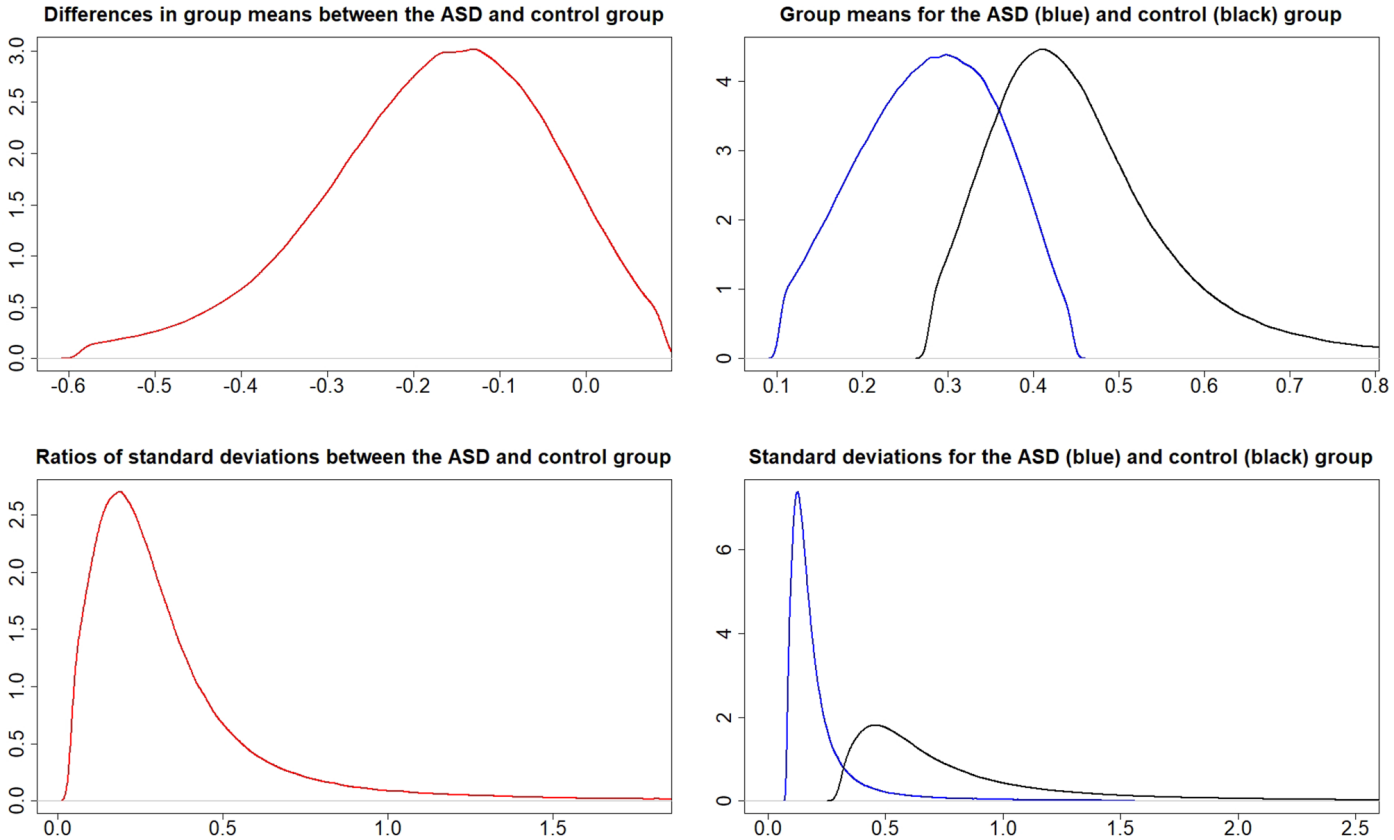
Posterior distributions of $\mu _{A}-\mu _{C}$ (top left), $\mu _{A}$ and $\mu _{C}$ (top right), $\frac{\sigma _{A}}{\sigma _{C}}$ (bottom left), and $\sigma _{A}$ and $\sigma _{C}$ (bottom right) for metal Pb.

**Figure 6:**
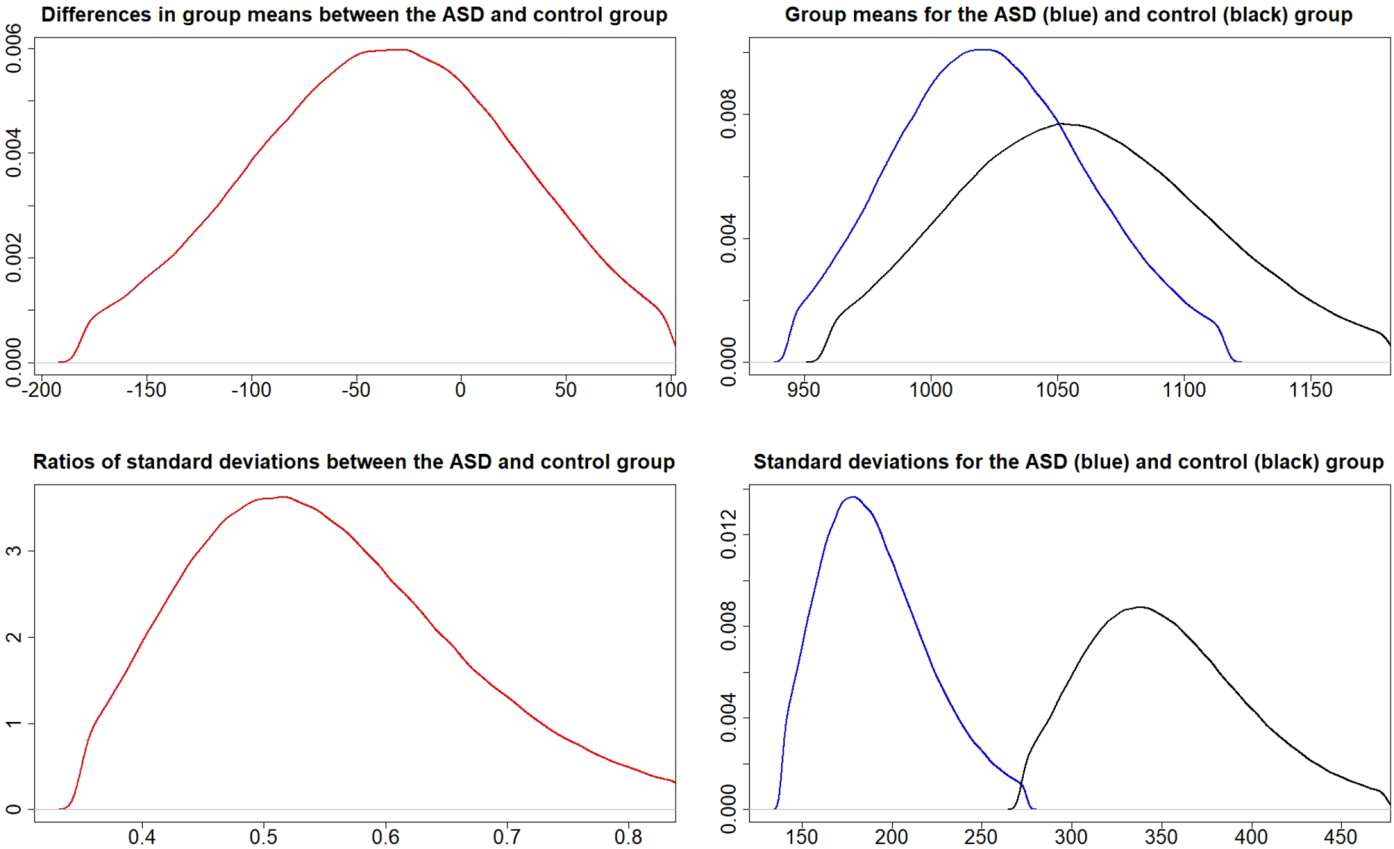
Posterior distributions of $\mu _{A}-\mu _{C}$ (top left), $\mu _{A}$ and $\mu _{C}$ (top right), $\frac{\sigma _{A}}{\sigma _{C}}$ (bottom left), and $\sigma _{A}$ and $\sigma _{C}$ (bottom right) for metal Zn.

Figures 1, 2, 3, 4, 5 and 6 show the posterior distributions of $\mu _{A},\mu _{C},\mu _{A}-\mu _{C}, \sigma _{A},\sigma _{C},\sigma _{A}/\sigma _{C}$ and the posterior probabilities $P\left( \mu _{A}>\mu _{C}\right)$, $P\left( \sigma _{A}>\sigma _{C}\right)$ for each of the metals Al, Cd, Hg, Li, Pb and Zn. To a large extent, the expected values $\mu$ and standard deviations $\sigma$ for metals Al, Hg, and Li, are higher in the ASD than in the control group. The opposite holds for metal Pb, where expected values and standard deviations tend to be higher for the control group, and for metal Zn, where standard deviations tend to be higher for the control group. No differences between the groups in expected values and standard deviations are apparent for metal Cd. However, visual inspections from the figures can be more precisely quantified and interpreted using the calculated probabilities $P\left( \mu _{A}>\mu _{C}\right)$ and $P\left( \sigma _{A}>\sigma _{C}\right)$ (shown in the figures). The posterior odds in favor of $\mu _{A}$ is given by

$$\begin{aligned} K=\frac{P\left( \mu _{A}>\mu _{C}\right) }{P\left( \mu _{C}>\mu _{A}\right) }. \end{aligned}$$

Analogously to the interpretation of the Bayes factor in^25^, we provide similar interpretations of *K* in Table 1.

**Table 1:** Interpretation of the posterior odds *K* in favor of either $\mu _{A}$ or $\mu _{C}$.

*K*	$P\left( \mu _{A}>\mu _{C}\right)$ or $P\left( \mu _{C}>\mu _{A}\right)$	Strength of evidence for $\mu _{A}>\mu _{C}$ or $\mu _{C}>\mu _{A}$
1 to 3.2	0.5 to 0.762	Not worth more than a bare mention (No)
3.2 to 10	0.762 to 0.909	Weak (Wk)
10 to 100	0.909 to 0.990	Strong (Str)
> 100	$> 0.990$	Decisive (Dec)

The probabilities for $P\left( \mu _{A}>\mu _{C}\right)$ and $P\left( \sigma _{A}>\sigma _{C}\right)$ from Figs. 1, 2, 3, 4, 5 and 6 can be extracted and combined with the interpretations of the posterior odds *K* from Table 1. Differences between the autism and control groups in $\mu$ and $\sigma$ for each metal are given in Table 2. There is strong evidence that the mean and standard deviation of Al are higher in the ASD group than in the control group. There is also strong evidence that the standard deviation of Pb is higher in the control group and that the standard deviation of Li is higher in the ASD group. There is decisive evidence that the standard deviation of Zn is higher in the control group compared to the ASD group.

**Table 2:** Probabilities and strength of evidence for the mean and standard deviation of the metal values in the cord blood, respectively, being higher in one of the groups.

Metal	$P\left( \mu _{A}>\mu _{C}\right)$	Strength of evidence	$P\left( \sigma _{A}>\sigma _{C}\right)$	Strength of evidence
Al	0.953	Str: $\mu _{A}>\mu _{C}$	0.961	Str: $\sigma _{A}>\sigma _{C}$
Cd	0.698	No	0.524	No
Hg	0.837	Wk: $\mu _{A}>\mu _{C}$	0.816	Wk: $\sigma _{A}>\sigma _{C}$
Li	0.750	No	0.981	Str: $\sigma _{A}>\sigma _{C}$
Pb	$1-0.895$	Wk: $\mu _{C}>\mu _{A}$	$1-0.932$	Str: $\sigma _{C}>\sigma _{A}$
Zn	$1-0.697$	No	$1-0.995$	Dec: $\sigma _{C}>\sigma _{A}$

**Figure 7:**
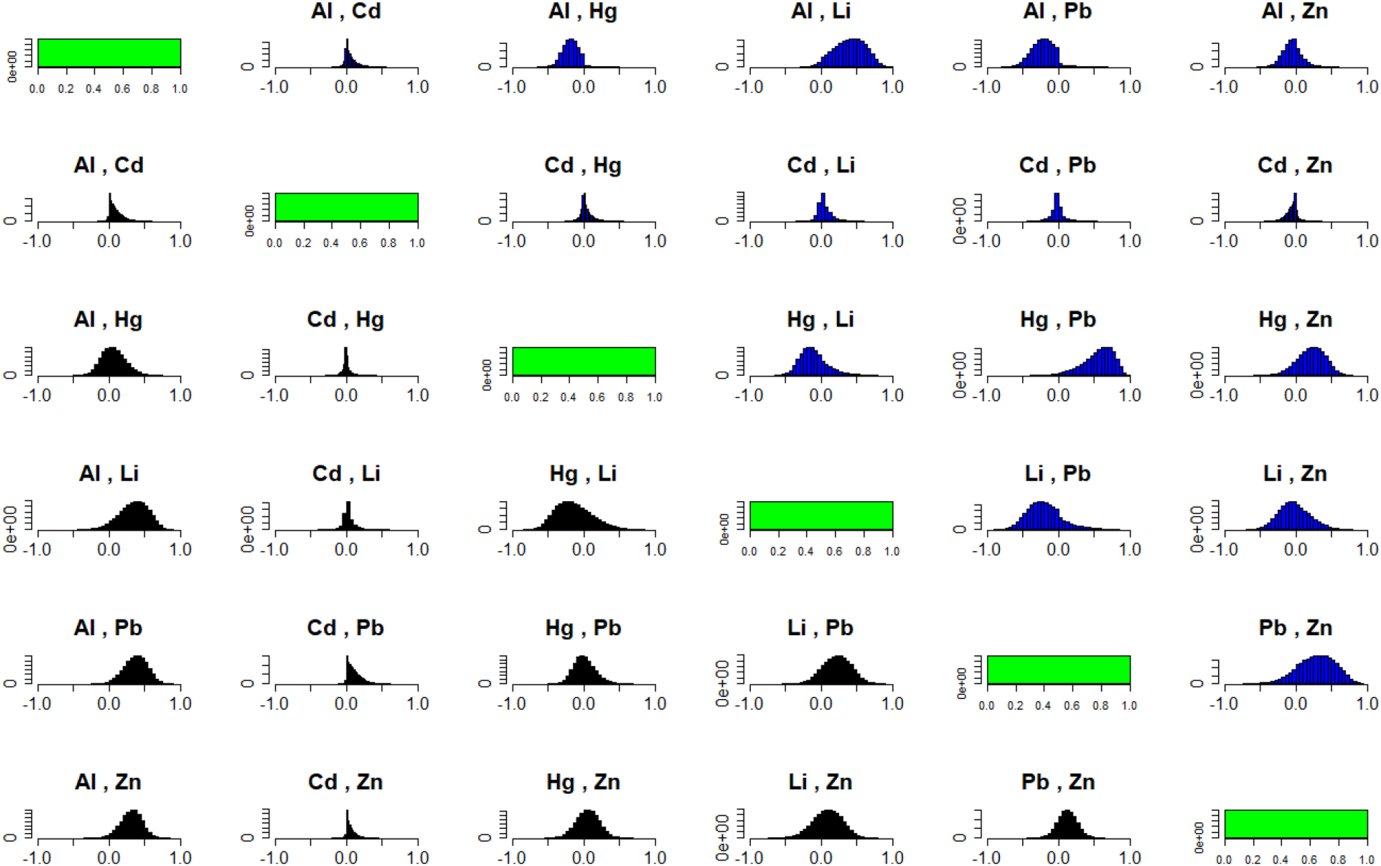
Histograms of posterior correlations between the metals for the control group (black) and ASD group (blue).

In order to identify if linear associations between the metals would be relevant for the ethiology of ASD, we performed inference on pairwise correlations of the metals. These were extracted from the posterior inference of the elements in $\Sigma$ for each group. Table 3 shows that especially Pb tends to be associated with other metals. There is decisive evidence that a linear association between Pb and Al is larger in the control group than in the ASD group. Likewise, there is strong evidence of a higher linear association between Pb and Hg in the autism group, and of a higher linear association between Pb and Li in the control group. In addition, there is strong evidence of a higher linear association between Al and Hg, Al and Zn, and Zn and Cd in the control group. Figure 7 confirms this view as the majority of the histograms of correlations for the control group are shifted more to the right compared to the corresponding histograms of the ASD group.

**Table 3:** Probabilities and strength of evidence for the pairwise correlations of metals being higher in one of the groups.

Metal	$P\left( \rho _{A}>\rho _{C}\right)$					
	Al	Cd	Hg	Li	Pb	Zn
Al		0.656	0.910	1-0.572	0.998	0.974
Cd	No		1-0.642	1-0.551	0.904	0.940
Hg	Str: $\rho _{C}>\rho _{A}$	No		1-0.555	1-0.980	1-0.755
Li	No	No	No		0.922	0.665
Pb	Dec: $\rho _{C}>\rho _{A}$	Wk: $\rho _{C}>\rho _{A}$	Str: $\rho _{A}>\rho _{C}$	Str: $\rho _{C}>\rho _{A}$		1-0.721
Zn	Str: $\rho _{C}>\rho _{A}$	Str: $\rho _{C}>\rho _{A}$	No	No	No	

### Metal values for 5-year olds

Table 4 shows a strong evidence for higher average metal values of Hg in the ASD group compared to the control group. Strong evidence also exists for the standard deviation being higher for Li in the control group and Zn in the autism group. No decisive evidence was found for any of the metals in 5-year olds.

**Table 4:** Probabilities and strength of evidence for the mean and standard deviation of metal values for 5-year-olds, respectively, being higher in one of the groups for each metal.

Metal	$P\left( \mu _{A}>\mu _{C}\right)$	Strength of evidence	$P\left( \sigma _{A}>\sigma _{C}\right)$	Strength of evidence
Al	$1-0.718$	No	$1-0.547$	No
Hg	0.988	Str: $\mu _{A}>\mu _{C}$	0.810	Wk: $\sigma _{A}>\sigma _{C}$
Li	$1-0.709$	No	$1-0.938$	Str: $\sigma _{C}>\sigma _{A}$
Zn	0.817	Wk: $\mu _{A}>\mu _{C}$	0.936	Str: $\sigma _{A}>\sigma _{C}$

### Group comparison between metal values in the cord blood and for 5-year olds

Table 5 shows decisive evidence for the means and standard deviations of almost all metal values being higher in 5-year-olds. One exception was Zn, where the evidence is decisive for both the mean and standard deviation being higher in the cord blood than for 5-year-olds. Another exception was Hg, where the evidence is only weak for both the mean and standard deviation being higher in 5-year-olds in the control group and where no evidence exists for the standard deviation in the ASD group being higher in either the cord blood or in 5-year-olds.

**Table 5:** Probabilities and strength of evidence for the mean and standard deviation of the ratios $R_{ASD}=\frac{X_{Aut}^{(5)}}{X_{Aut}^{(0)}}$ and $R_{Control}=\frac{X_{Control}^{(5)}}{X_{Control}^{(0)}}$ being higher for 5-year-olds compared to metal values in the cord blood, where $X_{Control}^{(0)}$ and $X_{ASD}^{(0)}$ denote the metal values in the cord blood and $X_{Control}^{(5)}$ and $X_{ASD}^{(5)}$ the metal values for 5-year olds in the control and ASD groups, respectively.

	Metal	$P\left( \mu _{R}>1\right)$	Strength of evidence	$P\left( \sigma _{R}>1\right)$	Strength of evidence
**ASD**	Al	1	Dec: $\mu _{R}>1$	1	Dec: $\sigma _{R}>1$
	Hg	1	Dec: $\mu _{R}>1$	$1-0.687$	No
	Li	1	Dec: $\mu _{R}>1$	1	Dec: $\sigma _{R}>1$
	Zn	$1-0.990$	Dec: $\mu _{R}<1$	$1-1$	Dec: $\sigma _{R}<1$
**Control**	Al	1	Dec: $\mu _{R}>1$	1	Dec: $\sigma _{R}>1$
	Hg	0.853	Wk: $\mu _{R}>1$	0.844	Wk: $\sigma _{R}>1$
	Li	1	Dec: $\mu _{R}>1$	1	Dec: $\sigma _{R}>1$
	Zn	$1-1$	Dec: $\mu _{R}<1$	$1-1$	Dec: $\sigma _{R}<1$

### Classification results

**Figure 8:**
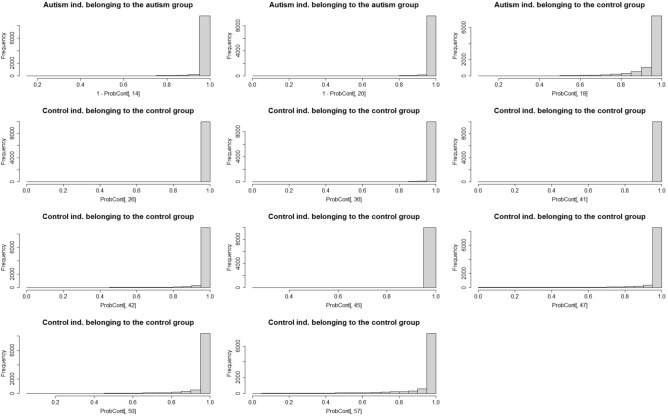
Histograms for the posterior probability that each actual ASD or control individual belongs to either the ASD or control group using 10000 posterior draws of $\left( \mu ,\sigma \right)$.

In order to evaluate the ability of our cord blood data to predict future ASD incidence, we performed an analysis of the strength of evidence for an observation to belong to either the ASD or the control group (as described in Section *Classification*). Figure 8 shows the posterior distributions (using 100000 posterior draws) of $p\left( C_{i}=c_{1}|y_{i},y_{-i}\right)$ for each individual *i*. Of the classified individuals, all but one were correctly classified to one of the groups. However, for this individual the average posterior probability, $\underset{\mu ,\sigma }{\text {mean }}p\left( C_{i}=c_{1}|y_{i},y_{-i}\right) =0.938$, is actually the lowest average probability for all the 11 individuals in the figure. The individuals in Fig. 8 show at least strong favor to one of the groups according to our following definition:

$$\begin{aligned} \underset{\mu ,\sigma }{\text {mean}} p\left( C_{i}=c_{1}|y_{i},y_{-i}\right) >0.909\Leftrightarrow \text {at least strong favor to group} C_{j}. \end{aligned}$$

The remaining 49 individuals can not be classified according to this definition (Table 6).

**Table 6:** Confusion matrix for the classified 11 individuals with at least strong favor to one of the groups.

Confusion matrix		Predicted class (classified)			Not classified
		Control	Autism	Total	
Actual class	Control	8	0	8	32
	Autism	1	2	3	17
	Total	9	2	11	49

When employing a more conservative rule, by changing the threshold above from 0.909 to 0.990, all 5 individuals were correctly classified with at least decisive favor to one of the groups, see Table 7.

**Table 7:** Confusion matrix for the classified 5 individuals with at least decisive favor to one of the groups.

Confusion matrix		Predicted class (classified)			Not classified
		Control	Autism	Total	
**Actual class**	Control	3	0	3	37
	Autism	0	2	2	18
	Total	3	2	5	55

### Posterior predictive distributions and posterior odds for each type of metal values

In order to predict possible metal values for a new individual, we obtained the posterior predictive distribution, $p\left( {\tilde{x}}|x\right) ,$ for future values ${\widetilde{x}}$ given observed values *x* for each type of metal in each of the ASD and control groups. Investigating group differences of the posterior predictive distributions further, we generalized the odds, defined in Section *Classification* for an individual *i*, to the following odds for any future value ${\tilde{x}}$ of a metal belonging to the control group:

$$\begin{aligned} O_{Control}|{\widetilde{x}},x=\frac{p\left( C_{{\widetilde{x}}}=c_{2}|{\widetilde{x}},x\right) }{p\left( C_{{\widetilde{x}}}=c_{1}|{\widetilde{x}},x\right) }=\frac{p\left( {\widetilde{x}}|x,C_{i}=c_{2}\right) }{p\left( {\widetilde{x}}|x,C_{i}=c_{1}\right) }. \end{aligned}$$

**Figure 9:**
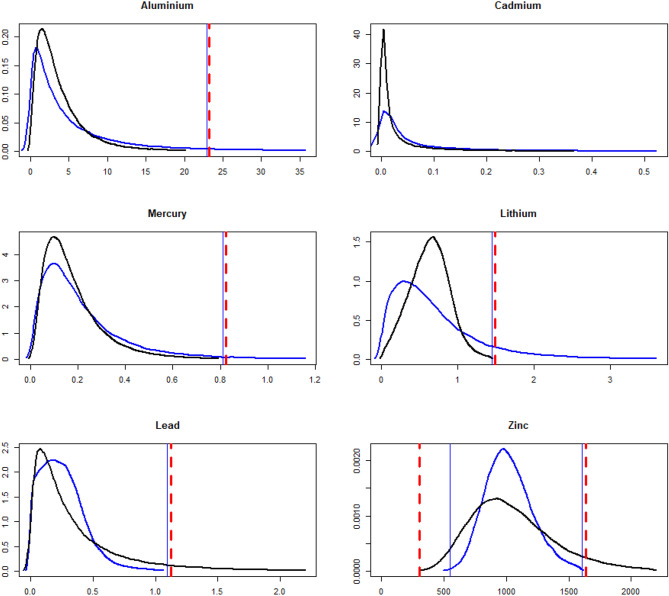
Posterior predictive distributions for each type of metal value in the control (black) and autism group (blue). The metal values *x* are simulated from taking the exponential of the multivariate normal realisations $\ln x$, conditional on the posterior draws of $\mu$ and $\Sigma$. The 1% most extreme metal values are not shown for each metal type due to poor visualization of the whole distribution from showing the characteristic thick and long tails in the log-normal distribution. The solid vertical lines show thresholds of strong evidence that an individual with more extreme values belongs to the ASD group for metals Al, Cd, Hg, Li, and to the control group for metals Pb and Zn. The dashed lines show corresponding thresholds of decisive evidence.

Hence, the odds for any metal value ${\tilde{x}}$ is given by the ratio of the posterior predictive densities. Figure 9 shows the posterior predictive distribution $p\left( {\tilde{x}}|x\right)$ for each type of metal and group. The distributions of the groups for each metal overlap to a certain degree. However, for all metals but Pb and Zn the distribution for the autism group attains, in general, larger values in long and thick tails of the distribution. Hence, an extreme value for each type of metal can possibly be used as a biomarker for an individual belonging to either group. By marking out thresholds of metal values in the tails of $p\left( {\tilde{x}}|x\right)$ in Fig. 9 that correspond to considerably low or high values of the odds $O_{Control}|{\widetilde{x}},x$, the more extreme metal values beyond each threshold can be used as a biomarker for an individual with such a value to belong to either group. Table 8 shows the values of the marked thresholds in Fig. 9.

**Table 8:** Threshold values for strong and decisive evidence of a metal value *x* belonging to the ASD (in bold) and control (in italics) groups.

Metal	Strong evidence	Decisive evidence
Al	$x>22.89$	$x>23.21$
Cd	−	−
Hg	$x>0.812$	$x>0.823$
Li	$x>1.448$	$x>1.485$
Pb	$x>1.093$	$x>1.123$
Zn	$x<547.5,\,x>1608.5$	$x<302.4,\,x>1633.6$

**Figure 10:**
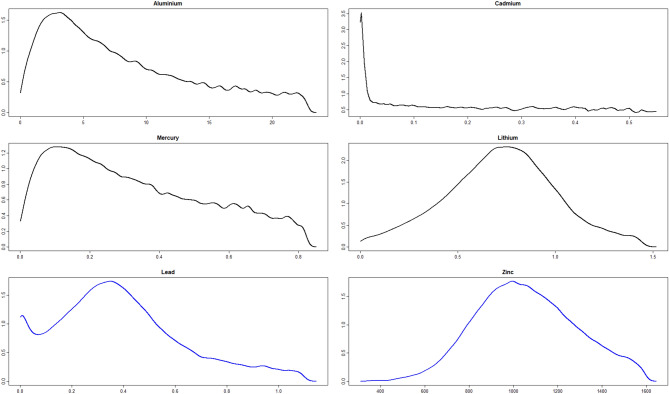
The posterior odds for each metal value belonging to either the control (black) or the autism (blue) group.

This complements the previous classification results in Tables 6 and 7, where the whole vector of metal values for an individual was used to classify each individual to either group. However, tail distributions are known to be sensitive to outliers in particularly small datasets. Therefore, we need to interpret such a biomarker with caution in our relatively small dataset and be aware of the fact that more data can change tail distributions dramatically. Figure 10 shows the posterior odds as a function of values for each type of metal. For large values of Al, Cd, Hg and Li, the odds for being in the control group declines towards 0. This is in contrast to large values of Pb and Zn, where the odds for being in the autism group declines towards 0 instead.

## Discussion

In this study, we investigate the presence of heavy metals in the cord blood of newborns and in the serum of the same children at five years of age. To identify the relevance of specific metals (in these two stages of development) for the etiology of ASD, we used a Bayesian multivariate log-normal modeling that accommodates censored values. In cord blood, we found strong evidence for higher Al levels in the ASD compared to the control group. The role of Al as a neurotoxic and neuroinflammatory agent, and its synergistic interaction with Hg, has previously been reported^26^. In addition, Al has been shown to be strongly linked to ASD, with the severity of behavioral symptoms being correlated to Al levels in the hair^27^. To date, however, no study has suggested that higher levels of Al in the cord blood can be indicative of future ASD diagnosis, as our data indicates. The potential of prenatal Al levels to indicate later ASD incidence needs to be further investigated.

**Figure 11:**
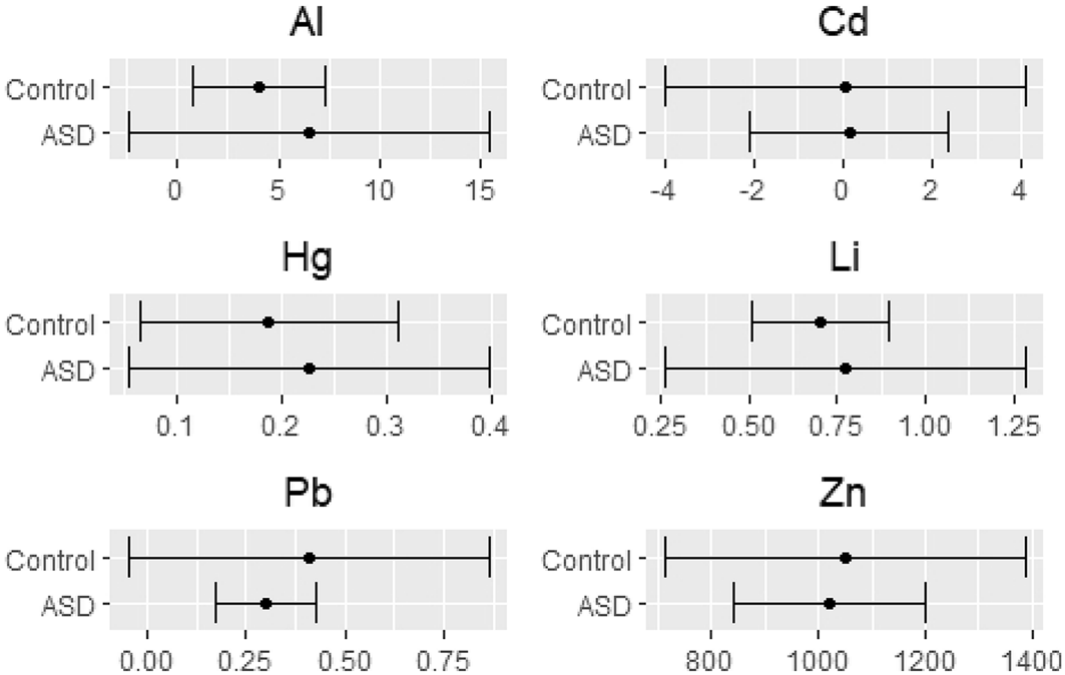
The interval ${\hat{\mu }}_{x}\pm {\hat{\sigma }}_{x}$ for each cord blood metal value in each of the ASD and control groups, where ${\hat{\mu }}_{x}$ and ${\hat{\sigma }}_{x}$ are the posterior modes as Bayesian point estimates from the posterior distributions of $\mu _{x}$ and $\sigma _{x},$ respectively.

We also found strong evidence for larger standard deviations of Al and Li in the ASD group, as well as for a larger standard deviation of Zn in the control group. Figure 11 summarizes our findings by using the posterior modes as Bayesian point estimates of the mean and standard deviation for each metal. For Al and Li this may indicate higher possibilities of a high, potentially toxic, exposure to these metals during pregnancy. To the best of our knowledge, no study has investigated the role of prenatal exposure to Li in relation to ASD. For Zn, in turn, our findings of decisive evidence for a higher standard deviation in the controls compared to ASD in cord blood samples suggests that the ASD population is exposed to less nutritional options providing Zn as a micronutrient than the control population. Zn is an important micronutrient whose deficiency during critical fetal periods is thought to contribute to the development of ASD^28^. Concordant with this idea, low hair Zn levels in infants are indeed associated with ASD^28^. Additionally, we found decisive evidence for higher average Zn levels in the cord blood compared to 5-year-olds in both experimental groups. Our combined evidence on Zn suggests the importance of Zn as a micronutrient during pregnancy and may indicate higher possibilities of a high, potentially beneficial, exposure to Zn during pregnancy in controls. Concordantly, less possibilities of higher Zn exposure during pregnancy in ASD might be indicative of lack of this nutrient in crucial early developmental stages. This hypothesis needs to be further examined.

We found decisive evidence for higher average levels of Al and Li in 5-year-olds compared to the cord blood, both in the control and ASD groups. This is expected, because as the children grew, they increasingly obtained these metals via nutrition. The exception was Hg, for which we found decisive evidence of an average increase in 5-year-old ASD children, but only weak evidence for an average increase in 5-year-old control children. This is concordant with previous research showing an impaired ability for eliminating Hg in relation to ASD. For example, newborns later diagnosed with ASD present a 7.7-fold reduction Hg levels in hair, which is one of the main pathways of metal excretion^29^. Such impaired ability to excrete Hg would lead to an increased accumulation in the body, which is concordant with a 1.9-fold increase in blood Hg observed in ASD-diagnosed subjects^30^. Another interesting observation in relation to Hg is that the standard deviation is never higher for 5-year-olds in the ASD group compared to merely weak evidence for controls. This provides further insight to the idea that the increase in Hg levels in 5-year-old ASD children only depends on their impaired metabolic ability to detoxify Hg and not on environmental availability.

Moreover, we investigated the ability of our cord blood data to predict future ASD incidence by using classification. Although only 11 individuals could be classified and 49 could not, all but one (belonging to the control group) was correctly classified. The predictive power from classifying specific cord blood metal levels can certainly be higher for a larger dataset and we believe this approach has good potential for early identification of susceptibility to develop ASD. This would be important to develop early interventions, e.g. dietary, to minimize exposure to certain metals in individuals with a higher risk of developing ASD later on.

Finally, by using posterior predictive distributions of the metal values, we identified threshold levels for specific metals that predict future ASD incidence. Threshold levels could be identified for all metals, except for Cd. For Al, Hg, Li and Pb threshold levels were identified for the right tail of the posterior predictive distribution, where large values for these metals are indicative of a future incidence of ASD. Although these levels are only based on our dataset, a similar approach can be employed in future studies that incorporate a larger sample of children to examine whether the levels reported here are reliable and how they can be refined. For Zn, we identified a potential non-monotonic effect, with values below and above thresholds in both the left and right tail of the predictive distribution being related to controls, rather than to ASD incidence. Although it is natural that higher cord blood levels of this important micronutrient would relate to the prevention of ASD, it is hard to understand how very low levels could do the same. Non-monotonic effects are a contentious topic in environmental toxicology, for which the mechanism and real implications are yet to be understood^31^. However, it is important to point out that in vitro non-monotonic effects have been reported for another metal, Copper, in Caco-2 cells^32^. In any case, our study points towards the feasibility of using levels of specific metals in the cord blood as a warning signal about future ASD incidence, and as previously mentioned, to prompt early interventions aimed at preventing or minimizing the detrimental effects of ASD in children. Such interventions could, for example, involve limiting exposure of susceptible individuals to metals such as Hg. There is accumulating evidence of Hg involvement in ASD, both from previous reports and in the present paper. Hg involvement in ASD seems to be via accumulation in the body^30^, which would in turn lead to disrupted neurodevelopment^9^. Thus, limiting Hg exposure/intake by susceptible individuals could in theory help to promote normal neurodevelopment during key developmental times, notably between birth and the age of six. It is during this age when human brain experiences dramatic changes in size, a 4-fold increase, and connectivity, involving processes such as neuronal arborization, synaptogenesis, glycogenesis and myelination^33^.

### Supplementary Information

Supplementary Information 1.

## Data Availability

All data analysed during this study are included in this published article as a supplementary file.
